# Self-reported knowledge, attitude and mental health status of in-school adolescents in Nigeria

**DOI:** 10.4102/phcfm.v17i1.4858

**Published:** 2025-04-10

**Authors:** Atinuke O. Olowe, Amme M. Tshabalala, Judith C. Bruce

**Affiliations:** 1 Department of Nursing Education, Faculty of Health Sciences, University of the Witwatersrand, Johannesburg, South Africa; 2 Department of Nursing Science, Faculty of Clinical Sciences, University of Lagos, Lagos, Nigeria

**Keywords:** knowledge, attitude, mental health, mental health status, in-school adolescents

## Abstract

**Background:**

The global rise in adolescent mental health conditions highlights the need for preventive interventions particularly in schools for timely access to young people, building on inherent strengths and competencies.

**Aim:**

The study aims to determine the knowledge, attitude, mental health status and the predictors of mental health status of in-school adolescents.

**Setting:**

The study was conducted in government-owned secondary schools in Lagos State, Nigeria. Simple random sampling was used to select one of three senatorial districts; one junior and one senior secondary school with a nurse-led school clinic were purposively selected from the sampled district.

**Methods:**

Within a cross-sectional survey design, a self-administered questionnaire was used to obtain data from a sample of in-school adolescents aged 10–19 years (*n* = 148), enrolled in junior and senior classes.

**Results:**

Most in-school adolescents reported poor knowledge (62.2%; *n* = 92) and poor attitude (54.7%; *n* = 81) towards mental health; 37.2% (*n* = 55) reported being substantially at risk of conduct problems. A high proportion (79.7%) indicated normal prosocial behaviours. Level of knowledge (odds ratio [OR] = 3.25; *p* < 0.05; 95% confidence interval [CI] = 1.34–7.86) as well as third or higher birth order (OR = 3.46; *p* < 0.05; 95% CI = 1.34–8.94) were significant predictors of mental health status.

**Conclusion:**

Most in-school adolescents lack knowledge, have a poor attitude towards mental health and are more likely to display conduct problems impacting their mental health status.

**Contribution:**

The study provides baseline evidence for designing in-school programmes with a mental wellness focus to promote the mental health of adolescents, leveraging professional and parental networks.

## Introduction

Adolescents tend to grow rapidly, learning, adapting and developing in neurobiological and formational ways. Adolescence, typically between the ages of 10 and 19 years, encompasses development in all spheres of life – physical, social, emotional and intellectual. Experiences of and the ability to cope with developmental changes differ among adolescents. Being unable to cope with or adjust to physical, social, mental and emotional changes puts adolescents at risk of mental health conditions.^1^ About half of mental health conditions start during adolescence, with an estimated 50% emerging before the 14th birthday; disruptive behaviour or conduct and anxiety disorders are leading manifestations of mental health difficulties.^2,3,4^

The worldwide prevalence of mental health disorders or conditions for a pooled sample of children and adolescents aged below 18 years is estimated to range between 10% and 20%.^5,6^ Globally, mental health conditions among adolescents account for 16% of the global burden of disease and injury,^7^ with suicide being the second leading cause of death.^8^ In line with the report of Schor,^8^ suicide is among the leading causes of death in United Nations Children’s Emergency Fund (UNICEF) regions such as Latin America and the Caribbean.^9^ The increasing prevalence of mental health conditions among adolescents is of great global concern, exacerbated by the coronavirus disease 2019 (COVID-19) pandemic in recent years^10^. With adolescents making up one-sixth of the global population, and almost a quarter in sub-Saharan Africa, urgency is needed to curb the impact of a rising prevalence particularly in low- to middle-income countries.^11^

There is a growing body of literature on adult mental health literacy. However, there are fewer studies on the mental health of children and adolescents,^12,13^ especially in Africa.^14^ Despite available evidence that about half of the new cases of mental health conditions start in mid-adolescence, there are limited health interventions to promote mental health and prevent mental health conditions in this age group.^15^ Having knowledge of mental health implies the ability to understand what mental health is, and when there is a shift from good to poor mental health; it may or may not include knowing ways to promote and sustain mental health. Knowledge acquisition usually focusses on maintaining positive mental well-being by developing competencies and positive help-seeking behaviour when there is any deviation from the normal way of life. Kutcher et al.^16^ and Nobre et al.^17^ report that good knowledge of mental health and mental health disorders, and a positive attitude reduce stigma and increase help-seeking behaviour.

Adolescents’ mental well-being, also known as mental health status, is apt for overall health. Mental health is defined as a state of mental well-being that enables people to cope with normal life’s stresses, realise their abilities, learn well and work well and contribute to community endeavours^18^; it involves optimal emotional, cognitive and social adaptability, and resilience, which is influenced by a combination of internal and external factors. In a bid to optimise mental well-being, positive mental health symptoms (strengths) and mental health problems (difficulties) as indicators of impending mental health conditions can be identified early for timely intervention. The Strengths and Difficulties Questionnaire (SDQ)^14^ is a useful tool to screen children and adolescents who are substantially, moderately or not at risk of having mental health conditions based on their reported mental health difficulties and competencies. Hence, prosocial attributes, conduct problems, hyperactivity, peer problems and emotional symptoms are the variables to be assessed when determining the mental health status of in-school adolescents.

The high level of needs for adolescents with mental health conditions in low and middle-income countries and the paucity of data prompted further research. This study aimed to determine the knowledge, attitude and mental health status of in-school adolescents in Lagos, Nigeria. The objectives of the study were three-fold: to determine in-school adolescents’ knowledge of and attitude towards mental health and mental health conditions; to establish the extent to which these mental health conditions are predictive of clinically significant mental health problems; and lastly, to assess adolescents’ mental health status based on their self-reported mental health strengths and difficulties.

## Research methods and design

### Study design

A descriptive, cross-sectional, multi-stage design was employed to collect self-reported data from a sample of in-school adolescents regarding their mental health status and their knowledge of and attitude towards mental health and mental health conditions.

### Setting

The study was conducted in two government-owned secondary schools in Lagos State in South-Western Nigeria. The East senatorial district was chosen among the three districts using a simple random sampling. Purposive sampling was used to select one junior and one senior secondary school that had a sick bay or a school clinic being managed by a Registered Nurse.

### Study population and sampling strategy

The target population included junior and senior school adolescents aged 10–19 years from junior class 1–3 and senior class 1–3 (*N* = 621). The sample size estimation was done using the formula by Charan and Biswas^19^ putting into cognizance the *p*-value of the study as 0.05, precision of 5% for type 1 error and the expected proportion based on a previous study carried out.^20^ Assuming a 20% non-response rate^21^ the sample size was estimated to be 160 (Equation 1).


$$\begin{array}{l} n={\text{Z}}_{1-}\alpha_{/2}^{2}\text{p}(1-\text{p})/d^{2}\\ n=1.96^{2}\times 0.096\;(1-0.096)/0.05^{2}\\ n=133+27\;(20\%\text{ attrition}).\\ n=160 \end{array}$$ [Eqn 1]

The purposively selected schools were stratified across both levels and classes to ensure adequate representation. The sample recruited from each grade level was proportional to the number of students in the total population. Using the class list as a frame, a sampling interval was calculated after randomly selecting the first element using a table of random numbers. Participants were recruited following written assent and consent from adolescents and parents, respectively; 148 participated, yielding a response rate of 92.5%.

### Data collection

The data-collection tool comprised of four components –three semi-structured questionnaires and one standardised questionnaire. Part one elicited participants’ socio-demographic data (12 items), which included age, gender, class, number of children in the family and the respondents’ position in the family (Table 1). Part two (29 items) comprised of a knowledge survey adopted from the Mental Health and High School Curriculum.^22^ A response of ‘Yes’ or ‘No’ was required; each correct answer scored ‘1’ and incorrect scored ‘0’ to give a total obtainable score of 29. Scores were expressed as percentages and categorised as ‘poor’ (≤ 49%), ‘moderate’ **(50–74) and ‘good’ (≥ 75)**. Part three (12 items) incorporated a 3-point Likert scale of ‘Disagree’, ‘Not sure’ and ‘Agree’ (1–3) to describe the attitude of adolescents towards mental health. The total obtainable score was 36, expressed as a percentage and categorised as ‘poor’ (≤ 49%), ‘moderate’ **(50–74) and ‘good’ (≥ 75)**. In the last part, the SDQ was used to determine the mental health status of in-school adolescents. The SDQ comprises five subscales: four focus on Difficulties (emotional symptoms, conduct problems, hyperactivity problems and peer relationship problems) and one on Strength (prosocial behaviour). Each subscale has five items rated against a 3-point Likert scale (0–2) as follows: 0 = ‘not true’, 1 = ‘somewhat true’ and 2 = ‘certainly true’. The Difficulties subscales altogether have a maximum obtainable score of 40 categorised as ‘not at risk’ (0–15), ‘slightly at risk’ (16–19) and ‘substantial risk’ (20–40). The Strength subscale has a maximum obtainable score of 10 categorised as ‘not at risk’ (6–10), ‘slightly at risk’ (5) and ‘substantial risk’ (0–4).

**TABLE 1:** Sociodemographic and family characteristics of respondents (*N* = 148).

Variables	*N*	%
**Demographic characteristics**		
**Age (in years)**		
10–12 (Early adolescence)	22	14.9
13–15 (Mid adolescence)	49	33.1
16–19 (Advanced adolescence)	77	52.0
**Gender**		
Female	56	37.8
Male	92	62.2
**Religion**		
Christian	111	75.0
Islam	37	25.0
**Class**		
Junior class (JSS 1–3)	64	43.2
Senior class (SSS 1–3)	84	56.8
**Position in the family**		
First	26	17.6
Second	53	35.8
Third or higher	69	46.6
**Family characteristics**		
**Father’ s education**		
None	3	2.0
Primary	1	0.7
Secondary	28	18.9
Tertiary	116	78.4
**Mother’s education**		
None	7	4.7
Primary	7	4.7
Secondary	43	29.1
Tertiary	91	61.5
**Parents’ employment status**		
Both employed	69	46.6
Both unemployed	12	8.1
One employed (father)	46	31.1
Single parent employed (mother)	18	12.2
Single parent unemployed	3	2.0
**Number of children**		
1–4	69	46.6
> 4	79	53.4
**Household income**		
< N500 000	60	40.5
N500 000–N999 999	58	39.2
≥ N1 000 000	30	20.3

JSS, junior school sample; SSS, senior school sample; *N*, total number of respondents; *n*, given as number for each variable respectively.

Using Cronbach Alpha, the reliability measures were acceptable at α = 0.617 for each of the domains: knowledge about mental health, attitude towards mental health issues and mental health status of adolescents.

### Data analysis

Data were entered in a Microsoft Excel spreadsheet and imported into the Statistical software package, ‘STATA’, version 15. The data were analysed at univariate, bivariate and multivariate levels. Socio-demographic and family socioeconomic characteristics of the study participants were described using percentage distributions, while means and standard deviations were used for the description of respondents’ scores on knowledge, attitude and mental health indicators. Chi-square was used to determine associations between reported mental health status, demographic and family characteristics. Significance was set at *p* < 0.05. Binary logistic regression was used to predict the relationship between independent (categorical) variables by selecting the predictive model for dichotomous dependent variables.

### Ethical considerations

Ethical approval was obtained from the Health Research Ethics Committee of the Federal Neuro-Psychiatric Hospital, Lagos State, Nigeria (FNPH/HREC/19/18), and the University of the Witwatersrand Human Research Ethics Committee (Medical) (Certificate number M190802). Permission was granted by the office of the Head Service and the Education District II of Lagos State to conduct the study in the selected secondary schools. Prior visits were made to the selected schools and permission was sought from the school principals. Information sheets were given to both parents and adolescents. Adolescents who participated in the study were those who had both signed assent forms by themselves to affirm their willingness to participate in the study and consent forms from their parents or guardians. Anonymity and confidentiality were maintained throughout the study using research codes during data collection. All students in the schools selected were referred to the school nurses.

## Results

The mean age of respondents was 14.43 years (standard deviation [s.d.] = 1.95). More than half (52.0%; *n* = 77) were between the ages of 16 and 19, predominantly male (62.2%; *n* = 92) and of Christian religion (75.0%; *n* = 111). The majority (56.8%; *n* = 84) were in the senior classes and the rest were in the junior classes. Considering adolescents’ family characteristics, most had a father and/or a mother with tertiary education at 78.4% (*n* = 116) and 61.5% (*n* = 91), respectively. Parental employment status varied with less than half (46.6%; *n* = 69) of adolescents reporting that both parents were employed. The majority of households (53.4%) comprised of more than four children; the highest proportion of participants (82.4%) were in the second, third or higher birth order in the family, implying that most of them had at least one older sibling (Table 1). More than half (*n* = 58; 59.5%) of the respondents reported their income as N500 000 and above. The rest (*n* = 60; 40.5%) reported their income as less than N500 000 per month. However, more than half of the families (*n* = 79; 53.4%) had large family sizes, with more than four children in the family while the rest had fewer than four children.

### Mental health knowledge and attitude

**FIGURE 1:**
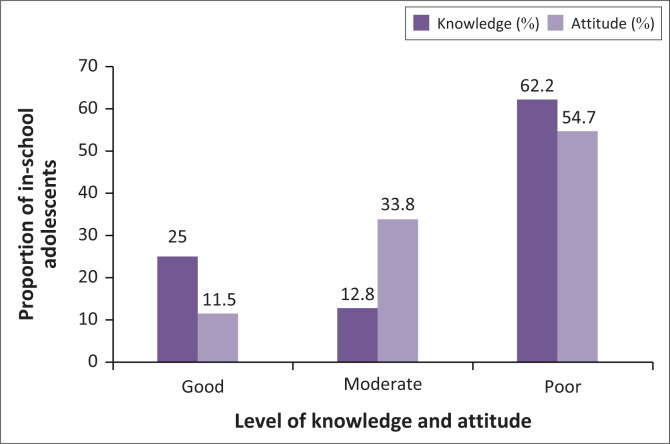
In-school adolescents’ knowledge of and attitude towards mental health.

Most in-school adolescents reported poor knowledge and attitude towards mental health and mental health conditions (62.2%; *n* = 92 and 54.7%; *n* = 81, respectively). A quarter (25%; *n* = 37) were rated as having good knowledge of mental health, and a lower percentage (11.5%) were rated as displaying a good attitude towards mental health and mental health conditions (Figure 1).

### Adolescents’ mental health status

In respect of mental health difficulties informed by the SDQ, most in-school adolescents considered themselves as ‘normal’ or not at risk for emotional symptoms (64.9%; *n* = 96), hyperactivity (69.4%; *n* = 102) and peer problems (45.3%; *n* = 67). More than one-third (37.2%; *n* = 55) of the adolescents indicated being substantially at risk of conduct problems. Overall, most in-school adolescents (43.5%) indicated that they are not at risk for difficulties associated with mental health conditions; 79.7% (*n* = 118) reported normal prosocial behaviour, which indicates a measure of mental health strength (Table 2). There were missing data in the case of ‘hyperactivity’ (*n* = 147), which reflected in the overall Difficulty score. There was no statistically significant association between mental health status and age (*p* = 0.952); class (*p* = 0.117); gender (*p* = 0.819); household income (*p* = 0.882); parental education (*p* = 0.860) and parental employment status (*p* = 0.663) except for adolescent position in the family, shown to be significant at *p* = 0.040.

**TABLE 2:** Reported mental health status of in-school adolescents.

Mental health status	Normal (Not at risk)		Borderline (Slightly at risk)		Abnormal (Substantially at risk)		Total *(N)*

	*n*	%	*n*	%	*n*	%	
Emotional symptoms	96	64.9	21	14.1	31	21.0	148
Conduct problems	54	36.5	39	26.4	55	37.1	148
Hyperactivity	102	69.3	22	15.0	23	15.7	147
Peer problem	67	45.3	43	29.0	38	25.7	148
Overall difficulty score	64	43.5	35	23.8	48	32.7	147
Prosocial behaviour	118	79.7	13	8.8	17	11.5	148

*N*, total number of in-school adolescent participants; *n*, number in-school adolescents for normal, borderline and abnormal mental status, respectively.

**FIGURE 2:**
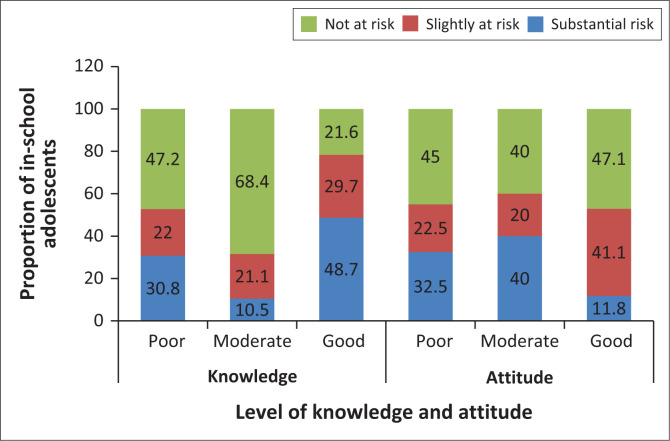
Proportion of risk of developing mental health problems by adolescents’ level of knowledge and attitude.

In determining the risk for mental health problems in relation to mental health knowledge, 48.6% (*n* = 72) of adolescents with good knowledge of mental health had a substantial risk of mental health problems. Approximately two-thirds (68.4%) with moderate knowledge of mental health reported not to be at risk of mental conditions; 47.1% of adolescents with good attitude towards mental health reported not to be at risk of mental health problems (Figure 2). There was a statistically significant association between mental health status and adolescents’ knowledge (*p* = 0.005) but not between mental health status and attitude towards mental health (*p* = 0.187).

### Predictors of mental health problems among in-school adolescents

Binary logistic regression was used to determine the association between adolescents’ knowledge of and attitude towards mental health, and the possibility of a clinically significant mental health problem. The unadjusted odds ratio (OR) in Model 1 shows that adolescents who reportedly have good knowledge levels have three-fold higher odds of being at risk of a clinically significant mental health problem (OR = 3.25; 95% confidence interval [CI] = 1.34–7.86) compared to their counterparts who had poor knowledge (reference group). Adolescents who were moderately knowledgeable were not significantly different from the reference group. The associations remain consistently significant when adjusting for other factors, including the adolescents’ involvement in physical activity, socio-demographics and family characteristics in Model 2 as shown in Table 3. Adolescents in the third or higher birth order (OR = 3.46; 95% CI = 1.34–8.94) had a more than three-fold higher odds of being at risk of clinically significant mental health problems than those who were the firstborn in the family. This association was consistent after adjusting for other factors in Model 2.

**TABLE 3:** Predictors of mental health problems among in-school adolescents.

Variables	Model 1			Model 2		
	Unadjusted OR	95% CI	Standard error	Adjusted OR	95% CI	Standard error
**Knowledge**						
Poor^R^	1.00	-	-	1.00	-	-
Moderate	0.41	0.14–1.18	0.222	0.40	0.13–1.28	0.237
Good	3.25	1.34–7.86**	1.465	3.88	1.45–10.42**	1.956
**Attitude**						
Negative^R^	1.00	-	-	1.00	-	-
Positive	1.23	0.60–2.51	0.449	1.58	0.70–3.59	0.662
Very positive	0.92	0.32–2.63	0.493	0.77	0.23–2.54	0.469
**Students’ socio-demographics**						
**Age**						
10–12-years^R^	1.00	-	-	1.00	-	-
13–15 years	1.39	0.72–2.70	0.470	2.65	0.67–10.48	1.860
16+ years	1.84	0.16–21.20	2.293	1.46	0.07–30.63	2.266
**Class**						
Junior class^R^	1.00	-	-	1.00	-	-
Senior class	1.27	0.66–2.45	0.427	0.60	0.15–2.48	0.435
**Gender**						
Female^R^	1.00	-	-	1.00	-	-
Male	0.85	0.43–1.67	0.292	0.91	0.42–1.95	0.353
**Position in the family**						
First^R^	1.00	-	-	1.00	-	-
Second	2.46	0.93–6.52	1.224	3.18	1.05–9.59*	1.791
Third or higher	3.46	1.34–8.94*	1.676	3.80	1.25–11.57*	2.158
**Religion**						
Christianity^R^	1.00	-	-	1.00	-	-
Islam	1.18	0.55–2.51	0.455	1.31	0.48–3.62	0.679
**Family socio-economic status**						
**Income**						
< N500 000^R^	1.00	-	-	1.00	-	-
N500 000–N999 999	0.84	0.41–1.75	0.314	0.83	0.33–2.08	0.389
≥ N1 000 000	1.10	0.45–2.70	0.503	0.51	0.15–1.76	0.321
**Parents’ education**						
Both no tertiary education^R^	1.00	-	-	1.00	-	-
One with tertiary education	0.68	0.24–1.89	0.355	0.63	0.19–2.08	0.383
Both tertiary education	1.05	0.42–2.64	0.494	1.34	0.43–4.16	0.775

^R^, reference category; CI, confidence interval; OR, odds ratio.

* , *p* < 0.05;

** , *p* < 0.01.

## Discussion

The mean age of in-school adolescents in this study was 14.43 years (middle adolescence), which falls within the predicted group at risk for developing mental health problems. Gleaning from prior studies, half of the mental health problems reported in the literature emerge during adolescent development with three-quarters emerging before 25 years of age and half before 14 years.^17,23,24^ Numerous negative outcomes are associated with the emergence of mental health conditions in this age group. In a longitudinal South African study commencing at the age of 14, Richter et al.^25^ found that adolescent mental health problems are associated with negative outcomes in areas such as education, employment, psychosocial functioning and interpersonal relationships. Age- appropriate and contextually relevant interventions^21^ are advocated to stem the tide of negative outcomes in later adulthood.

There are varying results from studies across regions regarding the effect of socioeconomic status on adolescents’ mental health. Some of these include parental educational level, parental employment status and family income. In addition, mental health inequities among adolescents because of their socioeconomic status, education and income reportedly affect their mental health negatively.^25,26^ Results from a longitudinal study done by Weinberg et al.^26^ showed that lower socio-economic status of the parents and education levels are associated with higher mental health issues and lower life satisfaction (happiness) among Dutch adolescents; parental socioeconomic status (inclusive of parental income) appears to be unrelated to adolescent mental health and life satisfaction.^26^ Similarly, although in a different context, this study showed no statistically significant association between adolescent mental health status and household income, parental education and parental employment status. This can be attributed to the fact that other variables apart from the ones mentioned can have the capacity to have an effect on the mental health status of adolescents including genetics, social relationships, personal experiences and the school environment.

Findings from this study suggest that adolescents who were third-born or later in their family were more likely to be at risk of significant mental health conditions compared to firstborns. Birth order is a variable that researchers rarely investigate; however, it is a significant variable during adolescent development. The impact of birth order on mental health is intertwined with biological conditions in utero, and social and environmental factors.^27^ In a British cohort study, though, Stannard et al.^27^ found no relationship between birth order and psychological distress in adulthood. Adolescents in the third or higher birth order were substantially at risk of clinically significant mental health problems than first-born adolescents in our study. Focussing on mid-life adults, a birth order of four or more and a low mental health score was found to be associated in men only.^27^ Similarly, a United Kingdom population-based, longitudinal study by Easey et al.^28^ showed that higher birth order increased the likelihood of mental health conditions in accordance with the findings of our study. In contrast, a cross-sectional study done on elementary school children in Japan investigating the association between birth order and mental health showed that early adolescents (10–13 years) with higher birth order were less likely to have mental health problems.^29^ This may be because of the lower age group and the likely presence of older siblings who can give social, emotional and protective cover for younger ones while growing up. In addition, differences in parenting practices among siblings also have an impact on child development and their mental health. Structured and shared parenting has benefits for both parent and child mental health, especially for younger children.^30^

Most in-school adolescents in our study reported poor knowledge of and attitude towards mental health. This is similar to the results of an Omani study on adolescents’ knowledge and attitude towards mental health in which 60% and 80% of students reported poor knowledge and attitude, respectively.^31^ However, a contrary finding was reported in India, where 60% of adolescents reported their knowledge level as average, and 12% above average.^32^ A natural response to poor mental health knowledge and attitude is to improve mental health education (literacy) in schools.^33,34^ Mental health literacy interventions inclusive of using the school curriculum are advocated as important for adolescents to improve knowledge and other competencies needed for them to stay mentally healthy and seek help in the event of mental health problems.^34,35^ While the evidence may be variable, studies have found these interventions improve knowledge scores^35,36,37^ but not necessarily attitude and health-seeking behaviour,^35^ except for a German study^37^ reporting a medium effect size for health-seeking efficacy. Noteworthy, is the relationship between knowledge and attitude whereby increases in knowledge significantly predict increases in positive attitudes towards mental health.^36^ Amid increasing mental health literacy research among adolescents, there are calls for a focus on knowledge of mental health knowledge rather than mental illness knowledge^38^ for impactful improvement in adolescent mental health.

Substantial risk of mental health problems was found among adolescents who reported good knowledge and attitude towards mental health. Although there are some limitations with self-report data, it is generally understood that having good knowledge of mental health does not automatically translate into effective coping skills and other ways of lowering risk. On the other hand, being knowledgeable about their mental health means that adolescents may be more likely to seek help on time compared to those without good knowledge and attitude. On the other hand, high expectations and pressure from the society, parents and self to be able to adapt and deal with stressful situations may lead to internalised stress and a greater risk for mental health conditions. There is limited research to support or refute our results. To appreciate the risk of mental health conditions, Renwick et al.^39^ suggest that more research is needed to understand the interplay between knowledge levels, beliefs and attitudes across varied cultural settings – studies in low-income countries are essential.^40^ Notwithstanding good knowledge, the characteristic nature of adolescents to engage in high-risk activities might jeopardise both their physical and mental health; experimenting with psychoactive substances and alcohol puts them at substantial risk of behavioural problems, social challenges, accidents and fatalities.^41,42,43^ Good mental health knowledge alone does not necessarily protect adolescents from mental health risks – mediating variables and protective factors, such as personal and social resources warrant further research to fully understand and reduce adolescents’ tendency to engage in risky behaviours.^41^

The mental health status of adolescents was determined by their reported difficulties and strengths according to five subscales in the SDQ.^14^ Mental health status, based on the total difficulties score at 43.5% (normal) was lower than in studies done in India (54%), South Africa (55%) and Scandinavia (83%).^44,45,46^ Comparison against results from the South African study^45^ takes into account the fact that the adolescent sample was selected from a hospital out-patient department. In determining the category of highest risk, about one-third had abnormal scores for conduct problems suggesting substantial risk for mental health problems – lower proportions between 13% and 15% were found in studies of school-going adolescents in similar contexts.^34,44^ Prosocial behaviour as an indicator of mental health strength was reported by the majority, which is in sharp contrast to other studies across mixed geographic and economic characteristics^34,44,45,46^ The SDQ was found to be a useful measure in several contexts but constraints in cross-country usage and comparisons are noted.^14,47^

### Strengths and limitations

This study involved in-school adolescents in two government-owned secondary schools from only one senatorial district in Lagos State. While this was beneficial for a better understanding of the associations between the variables, more schools should be involved – government-owned and private schools, to increase the generalisability of the study. Although participants were encouraged to answer the questions with sincerity, self-reports run the risk of bias because participants may provide responses that they believe are socially acceptable or correct according to the researcher’s expectations. Including teachers and parents to provide their versions may assist in substantiating adolescent reports or accounts.

### Recommendations

The school environment has consistently shown to be an appropriate space for mental health screening, monitoring, education and support of children and adolescents in different classes or education levels. Equipping adolescents with mental health knowledge and help-seeking skills is not enough unless it harnesses a professional approach and a wellness focus when providing education and services. It is recommended that the services of school nurses and/or primary care nurses be formalised in education settings for targeted mental health promotion strategies. The emphasis is on maintaining positive mental well-being and developing competencies that foster positive help-seeking behaviours. School nurses are best positioned to identify adolescents at risk through screening and monitoring, and for early interventions. Collaboration with teachers, parents and other health professionals to create a supportive network to address mental health holistically, is further recommended.

This study was conducted among in-school adolescents in South-Western Nigeria – similar studies from other regions in the country are recommended. This is necessary to leverage diverse perspectives because of each region’s unique cultural, social and economic contexts. Also, this will help researchers to develop targeted interventions by understanding specific challenges faced by in-school adolescents in different regions. Further research by way of longitudinal and cohort studies should examine the impact and outcomes of interventions to strengthen adolescent mental health. This should include evaluation and comparative studies of mental health education programmes that have been mainstreamed in the school curriculum or provided as standalone mental health courses.

## Conclusion

A significant gap in mental health knowledge and attitude among in-school adolescents was identified, which calls for an urgent need for health and educational interventions that focus on mental well-being as opposed to mental illness. Adolescents with high knowledge of mental health are predicted to be at higher risk of having a clinically significant mental health problem. This counterintuitive finding further accentuates the need for comprehensive approaches to equip adolescents with a range of life skills for mental well-being. While mental health education is crucial, the association between higher birth order and adolescents at risk highlights the role of family dynamics in optimising mental health among adolescents – here, the role of parents, guardians and family cannot be overemphasised.
